# Calculated Feelings: How Children Use Probability to Infer Emotions

**DOI:** 10.1162/opmi_a_00111

**Published:** 2023-10-27

**Authors:** Tiffany Doan, Ori Friedman, Stephanie Denison

**Affiliations:** Department of Psychology, University of Waterloo

**Keywords:** emotion attribution, probability, counterfactuals

## Abstract

Developing the ability to accurately infer others’ emotions is crucial for children’s cognitive development. Here, we offer a new theoretical perspective on how children develop this ability. We first review recent work showing that with age, children increasingly use probability to infer emotions. We discuss how these findings do not fit with prominent accounts of how children understand emotions, namely the script account and the theory of mind account. We then outline a theory of how probability allows children to infer others’ emotions. Specifically, we suggest that probability provides children with information about how much weight to put on alternative outcomes, allowing them to infer emotions by comparing outcomes to counterfactual alternatives.

## INTRODUCTION

Coming to understand others’ emotions is important in children’s social-cognitive development. This ability allows children to make sense of other people’s actions and to anticipate how their actions will affect others—it is at the heart of empathizing with and comforting others, but also with purposely surprising or intentionally irritating them. But how do children infer what others feel?

Initially, infants’ sensitivity to others’ emotions is evident from their ability to discriminate and recognize vocal and facial expressions. By 5 months of age, infants distinguish between positive and negative facial and vocal expressions (e.g., Barrera & Maurer, 1981; Walker-Andrews & Lennon, 1991), and by 7 months, they match positive and negative vocalizations to their respective facial expressions (e.g., Flom & Whiteley, 2014; Grossmann et al., 2006; see Ruba & Repacholi, 2020 for a review). At 10 months, infants can also match people’s emotional vocalizations to the probable causes of those emotions (e.g., Skerry & Spelke, 2014; Wu et al., 2017). At age 3, children recognize happy and sad facial expressions (e.g., Widen & Russell, 2003), and at 5, they recognize anger, fear, and surprise, though the ability to recognize these emotions improves with age (e.g., Gagnon et al., 2014; Guarnera et al., 2015). Across the preschool and elementary years, children become more adept at categorizing facial expressions (e.g., Plate et al., 2019; Woodard et al., 2022). For example, between the ages of 6 and 12, children become more flexible when updating their category boundaries of faces that were morphed from calm expressions to upset expressions (Plate et al., 2023). They also come to use people’s facial expressions to guide their own behaviors and to infer people’s mental states (e.g., Wu & Gweon, 2021; Wu & Schulz, 2018, 2020; Wu et al., 2021; also see Nook & Somerville, 2019 and Ruba & Pollak, 2020 for broader reviews of children’s emotional development).

Beyond recognizing emotions from facial expressions, with age, children also predict how others will feel by drawing on knowledge of how emotions are impacted by external events and by mental states (e.g., Lagattuta, 2005; Pons et al., 2004; Wellman & Banerjee, 1991; Widen & Russell, 2010, 2011). This additional ability allows children to infer how others feel when facial expressions are not available. For example, if a child learns that their friend did not make the softball team over text, she will not be able to use her friend’s facial or vocal expressions to infer how her friend feels. Instead, she has to rely on other information, such as whether her friend wanted to or expected to make the team, or her friend’s initial chances of making the team.

In this paper, we summarize recent work showing that children also infer emotions by drawing on their understanding of probability, specifically the probability of outcomes^1^, and offer a theoretical perspective on how this ability works. We review findings showing that with age, children increasingly recognize that emotions depend not just on events that happen, but on how these events relate to other things, including counterfactual alternatives that did not actually happen. We suggest that sensitivity to probability is crucial in these judgments because it helps children determine the weight or importance that should be given to different counterfactual alternatives.

**Figure 1.**
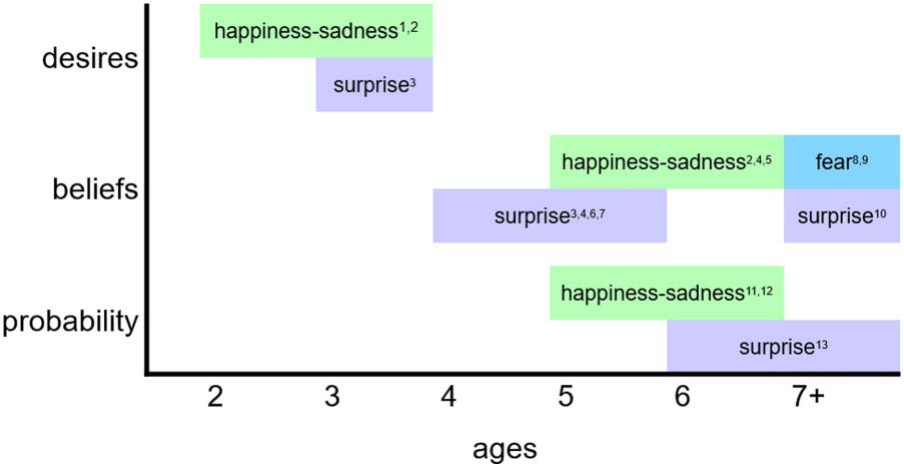
Developments in children’s use of theory of mind (desire and belief attributions) and probability to infer emotions. Works cited: [1] Wellman & Woolley, 1990; [2] Harris et al., 1989; [3] Wellman & Banerjee, 1991; [4] Hadwin & Perner, 1991; [5] Lara et al., 2019; [6] Wellman & Bartsch, 1988; [7] MacLaren & Olson, 1993; [8] Bradmetz & Schneider, 1999; [9] Ronfard & Harris, 2014; [10] Ruffman & Keenan, 1996; [11] Doan et al., 2020; [12] Doan et al., 2021; [13] Doan et al., 2018. *Note*. Surprise appears twice for beliefs because of a disagreement. Some researchers suggest children use people’s beliefs to infer surprise starting around ages 4 or 5. However, other researchers suggest these young children use ignorance rather than false belief, and that it is not until at least age 7 that false beliefs are used to infer surprise.

We first review two major accounts of how children infer emotions, namely by drawing on their knowledge of scripts and by using theory of mind. Then we review recent work on ways children consider probability when inferring emotions, and explain why these findings are not fully anticipated by the existing accounts. Specifically, we explain that while children do infer emotions in all of these ways, probability is sometimes needed to inform their reasoning about people’s mental states. Finally, we discuss the implications of the findings for children’s conceptions of emotions, and close by discussing avenues for future research. Figure 1 summarizes some of the major findings we review; the figure focuses on children’s use of theory of mind and probability to infer emotions.^2^

## ACCOUNTS OF HOW CHILDREN INFER EMOTIONS

One major account proposes that children understand emotions by learning and memorizing *scripts* (e.g., Widen & Russell, 2011). Scripts are knowledge structures consisting of sequences of specific, concrete events (e.g., Abelson, 1981; Schank & Abelson, 1977). They do not include abstract relations, nor do they provide an understanding of cause-and-effect. Instead, memorized scripts allow people to follow social conventions and to reason about common events. For example, ordering food at a restaurant may depend on knowing the script, *waiter brings menu, customer decides what to order, customer tells waiter, waiter tells cook* (Abelson, 1981). But again, the script only provides information about the order of events, and does not specify causal connections—it shows that the menu is brought before the customer decides what to order, but without specifying that receiving the menu allowed the customer to decide what to order.

With emotions, children may infer how people will feel by learning the emotions that typically follow from different sequences of events. That is, they can learn the types of situations that elicit each emotion and use it to infer people’s emotions in the future. Young children may initially start with just two broad scripts—one for positive emotions and one for negative emotions. With age, these scripts are differentiated until children have separate scripts for different positive and negative emotions (Widen & Russell, 2008, 2010, 2011). Broadly in line with the script account, preschoolers can predict that someone would feel a negative emotion (and not a positive one) if a negative event occurs, but they do not necessarily choose the correct negative emotion (e.g., predicting sadness instead of fear for fear-eliciting situations; Widen & Russell, 2011). With age, children’s improvement may result from increases in their knowledge of scripts for various emotions. For example, they predict that a person will be happy if they receive gifts, scared if they are chased by a dog, and angry if they were cut in line (e.g., Barden et al., 1980; Widen & Russell, 2010).

Another major account suggests that children infer emotions by drawing on their theory of mind. On this account, children anticipate how other people will feel by considering their mental states, including their desires and beliefs—that is, by considering what the other people *want* and *think* to be true^3^ (e.g., Harris, 2008; Lara et al., 2019; Wellman & Banerjee, 1991). For example, 3-year-olds predict that someone who receives a soda will be happy if they wanted one but will be sad if they instead wanted milk (Harris et al., 1989). Children also infer emotions by considering beliefs. They first use beliefs to infer happiness and sadness (e.g., Hadwin & Perner, 1991; Harris et al., 1989). For instance, 5-year-olds predict that a boy who likes candy will be happy if he *believes* a box contains some, even if it contains none (Hadwin & Perner, 1991). Later, around age 7, children also use beliefs to infer surprise (MacLaren & Olson, 1993; Ruffman & Keenan, 1996) and fear (Bradmetz & Schneider, 1999; Ronfard & Harris, 2014).

The script and theory of mind accounts are not mutually exclusive. Children infer emotions in both ways, and scripts and theory of mind each have strengths as means for inferring emotions. One strength of using scripts is that children do not need to know much about the particulars of a situation. For example, they can anticipate that the recipient of a gift will be happy, even if they don’t know what the gift is, or what the recipient wanted. Scripts may be especially useful for children with autism spectrum disorder. While children with autism struggle to use people’s mental states to infer their emotions, they can accurately infer people’s emotions by relying on concrete events in scripts (e.g., Baron-Cohen, 1991; Tan & Harris, 1991).

At the same time, using theory of mind allows for more flexibility for children who do not struggle with considering people’s mental states. One reason theory of mind provides this greater flexibility is because it relates mental states and emotions in an intuitive theory (e.g., Ong et al., 2015, 2019; Saxe & Houlihan, 2017; Wellman & Banerjee, 1991; Wellman & Woolley, 1990). Intuitive theories specify causal relations involving abstract constructs, allowing people to predict and explain outcomes (e.g., Gopnik & Meltzoff, 1997; Gopnik & Wellman, 2012). For example, children’s early theory of emotions may specify that having desires fulfilled causes people to be happy (for in-depth discussions of the intuitive theory of emotions see Anzellotti et al., 2021; Houlihan et al., 2023; Ong et al., 2019). If children were limited to only scripts, they would expect any given sequence of events to always end with the same emotional reaction. For example, heeding the script ‘*gift receipt is followed by happiness*’, would lead them to infer that people receiving gifts will always be happy. But sometimes gifts are disappointing. With theory of mind, children can ask how the gift compares with what the recipient wanted or expected.

## PROBABILITY

Beyond relying on scripts and theory of mind, children may also infer emotions by considering probability, specifically the probability of outcomes. For instance, their predictions about how a person will feel about an outcome may often depend on whether the outcome is likely or unlikely. This proposal may seem counterintuitive. After all, probability and emotions likely reflect distant conceptual domains. Probabilistic reasoning may reflect domain-general abilities that broadly shape children’s learning about the world (Denison & Xu, 2019; Xu et al., 2009). Meanwhile, children’s concepts of emotions may rest on more domain-specific knowledge about social cognition or possibly “theory of mind”.

Nonetheless, it is plausible that probability comes to be integrated in children’s emotion inferences. First, probability appears to be linked with naïve psychology in infancy (Wellman et al., 2016; Xu & Denison, 2009). Looking-time experiments show that when 11-month-old infants are familiarized to an experimenter’s goal of obtaining white rather than red balls, they expect her to sample white and not red balls from a population, even if the population mostly has red balls. However, if she cannot see the population from which she is sampling, then infants expect her to retrieve mostly red balls, in line with the population’s base-rate. Infants and children also use base-rates to predict outcomes, make inductive generalizations, and guide their decisions (e.g., Denison & Xu, 2014; Gweon et al., 2010; Téglás et al., 2011; Yost et al., 1962).^4^

Second, probability influences toddlers’ and preschoolers’ explicit social inferences (e.g., Gualtieri et al., 2020; Kushnir et al., 2010; Ma & Xu, 2011). By 20 months of age, children use probability to infer another person’s preferences. For example, when a person pulls a few ducks from a box containing duck and frog toys, children infer a preference for ducks if the box contains mostly frogs, but not if the box contains mostly ducks (Kushnir et al., 2010). This inference relies on probability. A person could randomly grab a small handful of ducks from a population of mostly ducks, but grabbing only ducks from a population of mostly frogs is improbable and thus implies a preference for ducks. Given that probability influences preference attributions in toddlerhood and is linked with naïve psychology in infancy, it may also be linked to emotion attributions.

In the remainder of the paper, we review evidence that young children come to use probability to infer emotions, and suggest these inferences are *not* fully captured by the script and theory of mind accounts. On first thought, this proposal might seem narrow. Although reasoning about probability is important, it is just one among many capabilities evident from early in development. Further, even if its influence is not fully captured by the script and theory of mind accounts, this is also likely true for other factors children consider when inferring emotions (e.g., agency, controllability, morality, ownership; Graham, 1988; Krettenauer et al., 2008; Pesowski & Friedman, 2015; Thompson, 1987; Yirmiya & Weiner, 1986). So why focus on probability specifically? We will suggest that one answer is that it is unique among factors because it is crucial for inferences about emotion based on counterfactual comparisons (Beck et al., 2014).

Before proceeding, it is important to clarify that in claiming that children use probability to infer emotions, we are not denying that they also use scripts and theory of mind. Indeed, it is difficult to think of any use of probability to infer emotions that would not require at least some use of theory of mind. For instance, to anticipate that someone’s emotional reaction to an event depends on its probability, children likely need to infer the person *knows* whether the event happened or not. The principal claim, then, is that children use probability over-and-above theory of mind and scripts. With age, children may incorporate probability into their intuitive theory of emotion. Just as children have a causal understanding of relations between mental states and emotions (e.g., Rieffe et al., 2005; Wellman & Banerjee, 1991; Wellman & Woolley, 1990), they might also see probability as causally relevant for emotions.

### Surprise

The link between probability and surprise seems intuitive. Indeed, adults think that improbable events are surprising. For example, people rate rainfall as more surprising when the prior likelihood of rain was low rather than high (Maguire et al., 2011), and people’s surprise about winning a gamble increases as their prior odds of winning decreases (Juergensen et al., 2014; also see Doan et al., 2023). Until recently, research on children’s understanding of surprise did not examine probability. Instead, research focused on beliefs—children’s understanding that surprise results when people discover their beliefs are false (e.g., Ruffman & Keenan, 1996; Scott, 2017). Children’s ability to use beliefs to *infer* surprise develops relatively late. Only children aged 7 and older consider people’s false beliefs when inferring their surprise (Ruffman & Keenan, 1996). Even so, children connect belief and surprise at younger ages when they are not predicting emotions. For example, when asked why a boy is surprised that his grandmother’s house is purple, 4-year-olds sometimes explain, “he didn’t *think* it would be purple” (Wellman & Banerjee, 1991).

Nonetheless, children also use probability to infer others’ surprise. In one experiment, children watched scenarios where two girls each received a red gumball (Doan et al., 2018). The gumballs were dispensed by machines that differed in their proportion of red and black gumballs. When asked which girl was more surprised about receiving a red gumball, 7-year-olds correctly judged that the character with the lower chance of receiving the red gumball was more surprised, 6-year-olds trended in this direction, 5-year-olds were at chance, and 4-year-olds incorrectly judged that the character with the higher chance of receiving the red gumball was more surprised. These findings suggest that with development, children come to use probability to infer other people’s surprise.

However, instead of directly using probability to infer surprise, children may have used probability to determine the character’s beliefs, and then used beliefs to infer surprise. For instance, children may have reasoned as follows: The girl who used the mostly black machine *believed* she would get a black gumball; and when the outcome contradicted her belief, she was surprised. Some previous findings suggest that children link surprise with belief from age 4. For example, 4-year-olds refer to people’s beliefs when explaining their surprise (Wellman & Banerjee, 1991), and some studies suggest that 4–5-year-olds attribute surprise to people who discover their beliefs are false (Hadwin & Perner, 1991; Wellman & Bartsch, 1988; also see MacLaren & Olson, 1993; but see Ruffman & Keenan, 1996). So it might seem that 7-year-olds could have succeeded in the gumball task by using the character’s beliefs to infer surprise, and 4- to 6-year-olds should have succeeded by doing the same. This possibility might seem to undermine the claim that 7-year-olds used probability to directly infer surprise – perhaps they instead succeeded by using probability to infer belief, and then used belief to infer surprise.

Follow-up experiments addressed these possibilities and concerns (Doan et al., 2018). In one experiment, children were asked a “prompt” question before the machines dispensed gumballs. In one condition, this prompt question required children to consider the characters’ beliefs about which color gumball they would receive; in another condition, the prompt required children to consider the likelihood of the characters receiving a gumball of a certain color; and in a control condition, the prompt did not require children to think of either beliefs or probability. Following the prompt, each machine dispensed a red gumball, and children were asked about which character was surprised. Despite all children answering the prompt questions correctly, children aged 5 were not influenced by the prompt questions, and they responded at chance when indicating which character was surprised to receive a red gumball. Children aged 6, however, responded differently depending on what they were prompted to think about. Those who were prompted to consider probabilities correctly judged that the character who was less likely to receive a red gumball would be more surprised to receive one. But children who were prompted to consider beliefs performed at chance, as did children who were asked a control prompt question. A second experiment replicated these findings in 6-year-olds but using a different task in which children only considered one agent.

The findings from these “prompting” experiments show that children link probability and surprise before age 7, they just don’t do so spontaneously. The findings also show that children do *not* need to consider other people’s beliefs to infer they will be surprised. In fact, considering other people’s beliefs did not even help children infer surprise! Despite correctly answering which character believed they would get a red gumball, children did not infer that that character would be surprised when they didn’t get one. This is puzzling given previous evidence that children link false belief and surprise (e.g., Hadwin & Perner, 1991; MacLaren & Olson, 1993; Wellman & Banerjee, 1991; Wellman & Bartsch, 1988). We suspect the conflict between these findings arises because, although children use beliefs to explain surprise at age 4, they may not infer surprise from belief until they are older. This conjecture is consistent with earlier findings suggesting that before children are 7, they use ignorance, rather than belief, to infer surprise (Ruffman & Keenan, 1996). Together, the findings from these surprise studies provide evidence that children can infer other people’s surprise by using information about probability (above-and-beyond any contribution of mental state reasoning).

### Happiness

Probability also influences people’s happiness. When adults win a gamble, they are happier if their chances of winning were particularly low (Mellers et al., 1997). Similarly, when a negative event occurs, adults are sadder when there was a lower chance of the negative event occurring (Shepperd & McNulty, 2002). Given that probability influences people’s own happiness about events, probability could also be used to infer others’ happiness. As with surprise, though, research on children’s understanding of happiness had not examined the influence of probability until recently. Instead, the primary focus was again on children’s understanding of relations between happiness and mental states (e.g., Hadwin & Perner, 1991; Harris et al., 1989).

But children do use probability to infer others’ happiness. One indirect indication that they do so comes from a study where children saw two bowlers attempt to knock down six pins (Asaba et al., 2019). Although both bowlers knocked down three pins, 5-year-olds thought one bowler would be happier than the other—they expected greater happiness from a bowler whose ball started poorly (it initially headed for the gutter) than from a bowler whose ball started well. Children likely based these inferences on the bowlers’ expectations—one bowler exceeded their expectations while the other bowler fell short of them (also see Lara et al., 2019). But probabilistic inferences may have contributed to children’s judgments. For example, the bowler initially on the path towards the gutter thought her ball would miss the pins because this was the most probable outcome (i.e., given the ball’s trajectory).

More direct evidence of children using probability to infer happiness comes from experiments that manipulated probability (Doan et al., 2020, 2021). In one set of studies, 4–6-year-olds watched a scenario where a girl received two yummy and two yucky gumballs from a machine (Doan et al., 2020). From age 5, children judged she was less happy with this outcome if the initial odds of getting yummy gumballs were high rather than low. In other studies, 6-year-olds’ judgments about how a girl would feel about winning an ordinary balloon depended on her odds of receiving a more attractive special balloon (Doan et al., 2021). They thought she would be more disappointed when the odds of winning a special balloon were high compared to low, and 5-year-olds also inferred this under some circumstances. Crucially in these experiments, children could not have inferred the girl’s happiness by *only* considering her mental states as the girl’s prior expectations were identical across conditions—she only learned the odds of winning a special balloon after she won the ordinary one. So, children’s inferences of happiness also depended on their probabilistic reasoning. They considered the odds of better or worse outcomes—that is, the odds of counterfactual alternatives.

### Relation to Script Theory and to Theory of Mind

Could children’s probability-based inferences result from their use of scripts or theory of mind? With scripts, we think the answer is *no*. Scripts are sequences of concrete events and outcomes (e.g., Abelson, 1981; Schank & Abelson, 1977). So probabilistic information cannot be incorporated into scripts: probabilities are not specific events and probability is abstract rather than concrete. For example, children could not use a script stating, “when unlikely events happen, people are surprised” as this would not be a script. Instead, it would be a principle in an intuitive theory of emotions (e.g., Houlihan et al., 2023; Ong et al., 2015, 2019; Smith-Flores & Powell, 2023). Also, script-based understanding was originally intended to explain how people come to make sense of situations they have previously experienced (e.g., see Section 3.7 in Schank & Abelson, 1977). It is unlikely, though, that children who participated in the experiments on probability-based inferences of emotions had experienced the specific situations they were asked about.

With theory of mind, the answer is less straightforward. For surprise inferences, it might seem that children could have reasoned as follows: the girl whose machine mostly contains black gumballs *believed* she would get a black one, so she is surprised when she gets a red one instead. But some findings undermine this belief-based account. As we reviewed above, in the experiment with prompts (Doan et al., 2018), some children were initially asked which girl *thinks* she will get a red gumball. While 6-year-olds answered this prompt correctly, it did not improve their ability to infer surprise. Thus, whereas prompting children to consider probability improves their inferences of surprise, prompting them to consider beliefs does not. This suggests that children’s inferences of surprise hinged on reasoning about probability above-and-beyond any contributions of mental state reasoning.

That said, children clearly had to draw on their theory of mind when inferring happiness, as when taking into account the girl’s preference for yummy gumballs over yucky ones. However, given that the girl’s preferences were identical across conditions, differences in children’s happiness ratings could not be attributed to their use of desires alone—they had to have also considered the probability of getting yummy and yucky gumballs. Children also could have considered beliefs and prior expectations—they might have recognized that when using a machine with mostly yummy gumballs, the girl would expect to get more yummy ones than yucky ones. Nevertheless, children had to consider probability as well. They could not infer that the girl would expect to receive more yummy than yucky gumballs unless they understood that the distribution of gumballs in the machine shapes the likelihood of different outcomes. So in the case of happiness judgments, it is unclear whether probability directly influences children’s inferences, or whether it only influences happiness inferences indirectly by allowing children to infer beliefs, and then use beliefs to infer happiness. The results from the gumball task (Doan et al., 2020) are compatible with both accounts. However, as noted above, with the balloons task (Doan et al., 2021), children could not have used the girl’s prior expectations to infer her emotions, as her prior expectations were the same across conditions. This suggests that children can use information about the probability of outcomes to inform people’s mental states in order to infer their happiness, but children can also use probability to directly infer people’s happiness, above-and-beyond the use of mental states.

## WHY PROBABILITY MATTERS: FACILITATING COUNTERFACTUAL COMPARISONS

As we have reviewed, children consider probability when inferring happiness and sadness at age 5; they can be prompted to use probability to infer surprise at age 6; and they spontaneously consider probability when inferring surprise at age 7. This developmental path suggests that with age, children understand that emotions do not depend just on raw outcomes (for corroborating evidence see Nook et al., 2020). Children instead increasingly view emotions as also depending on how outcomes *relate* to other things. When using theory of mind to infer emotions, children consider outcomes in relation to people’s mental states. With probability, children consider outcomes in relation to alternative possibilities. For example, when judging whether a girl would be happy with a balloon she won, children consider not only the balloon she received, but also the other available balloons.

Children’s probability-based inferences of emotion may therefore depend on considering outcomes in relation to counterfactual alternatives—potential outcomes that did not happen but could have. That is, information about the probability of outcomes may help children better see the causal relation between people’s actual outcomes, what they could have gotten, and their emotions. Indeed, adults’ probability-based judgments of emotions are typically interpreted in terms of counterfactual reasoning (e.g., Bell, 1985; Loomes & Sugden, 1986; Mellers et al., 1997; Shepperd & McNulty, 2002; van Dijk & van der Pligt, 1997), and young children are quite adept at reasoning about counterfactuals (e.g., Beck et al., 2006; Engle & Walker, 2021; German & Nichols, 2003; Kominsky et al., 2021; Nyhout & Ganea, 2019; Wong et al., 2023; also see Gautam & McAuliffe, 2023; though for findings suggesting later development of counterfactual-based inferences of emotions, see Beck & Crilly, 2009; Guttentag & Ferrell, 2004; Johnston et al., 2022). Further, children with autism can reason counterfactually (e.g., Scott et al., 1999) and in some cases, can use it to infer other people’s emotions (e.g., Begeer et al., 2014), suggesting a fruitful way for those who struggle with belief attributions to infer people’s emotions.

But how does probability matter for counterfactual comparisons? To illustrate this, consider how you would feel if you lost a contest that you had almost no chance of winning. Although you’re free to dwell upon the counterfactual outcome of having won, this might be unlikely given how bad your odds were. On the other hand, if winning was very probable and seemed assured, thoughts of having won might be difficult to avoid. As this example—and the reviewed findings—illustrate, probability determines (at least in part) how much weight children give different outcomes when comparing them. For example, we saw this in children’s understanding that receiving an ordinary balloon is only disappointing when the prior odds of a special balloon were high.

With surprise, weightings may be more complicated. In many situations, surprise may require both that the actual outcome was unlikely, and that some counterfactual alternative had seemed much more likely. After all, although we might be surprised to get the only red gumball from a machine that otherwise only contains blue ones, this would not be surprising if *all* the gumballs in the machine were unique. Again, surprise may require that both the actual outcome was improbable and that some unrealized alternative was instead statistically favored. Although, whether children draw this distinction has not yet been investigated, the prior findings nonetheless showed that they considered probability when inferring surprise.

Looking beyond children’s understanding of emotion, the findings also speak to the importance of probabilistic reasoning for children’s social cognition more generally. For instance, toddlers and young children consider probability when inferring people’s intentionality and preferences (e.g., Kushnir et al., 2010; Lopez & Walle, 2022). One way children do this is by assessing whether a person’s choices between objects are likely to reflect preferences, or could instead reflect random selection (Kushnir et al., 2010; also see Diesendruck et al., 2015). Children also consider a person’s choices between probabilistic options to determine what the person wanted (Doan et al., 2022). Recent work also shows that children use probabilistic information to make inferences about social relationships, norms, and evaluations (e.g., Eason et al., 2019; Heck et al., 2021; Hurst et al., 2020; Partington et al., 2023; Sehl et al., 2023; Vélez & Gweon, 2020). Further, in discussing how probability impacts happiness, we also noted that children may use probability to infer others’ beliefs—they may recognize that a girl who is likely to get yummy gumballs will expect to get them. The overall picture, then, is that young children’s judgments about the social and mental worlds are informed by probabilistic reasoning.

## FUTURE DIRECTIONS

Of course, much remains to be discovered about how children use probability to infer emotions. We have suggested that children use both theory of mind and probability to infer others’ emotions. This raises the question of whether these are truly distinct contributors to children’s emotion inferences. Alternatively, children might draw on some form of reasoning that fuses both kinds of information—for example, theory of mind representations that encode probabilistic information. Adults have such representations. They readily understand that people believe things with different degrees or probabilities of belief. That is, another adult can easily represent that when I can’t find my phone, I will generate a number of possibilities for where it might be and I likely hold different degrees of belief for each one. For example, I might think it’s pretty likely that I left it in the kitchen or my car, whereas having left it in the laundry room is possible but less likely (for experimental evidence of this and more sophisticated theory of mind inferences that involve probabilistic degrees of belief see Baker et al., 2017; Birch & Bloom, 2007). It would certainly follow that I’d then be more surprised to find it in the laundry room than the kitchen.

However, the balance of evidence so far suggests that children may not have such representations—mental state and probability information may be separate in them. First, children do not appear to incorporate probabilities into their belief attributions. Before ages 8 or 9, they do not seem to grasp that other people see some beliefs as very likely to be true and other beliefs as less likely to be true (Pillow, 2002; Pillow & Anderson, 2006; Pillow & Pearson, 2012; Pillow et al., 2000). Further, while prompting children to consider the probability of outcomes impacted their surprise inferences, prompting them to consider people’s beliefs did not (Doan et al., 2018). This suggests that children treat probability and beliefs as two different kinds of information. Future work can test this possibility directly. For instance, future work can investigate whether children rate the degree of probability and the degree of someone’s beliefs similarly (i.e., would an 85% chance of getting a red gumball equate to a person being 85% sure that they would get a red gumball?).

Relatedly, another unanswered question discussed above is whether children use probability to directly infer other people’s emotions or whether they use probability to inform people’s beliefs and expectations which are then used to infer emotions. It is clear in the case of surprise that children can directly use probability to infer surprise, but it is less clear in the case of happiness. For example, in the gumball task where a girl gets yummy and yucky gumballs from a mostly yucky machine (Doan et al., 2020), it is unclear whether children think the girl is happy because she had *high odds* of getting mostly yucky gumballs or because she *expected* to get mostly yucky gumballs. In other words, did children *only* think about the probability of getting the gumballs when inferring the girl’s happiness? Or did children use probability to infer the girl’s expectations, and then use those expectations to infer her happiness? To test between these possibilities, children could be prompted to consider probability or expectations before their emotion inferences. This would allow us to see if one improves their inferences more than the other. Another potential method is to ask children to explain why they think the girl feels a particular way—would they explain her emotions by referring to the probabilities of the possible outcomes, or would they explain her emotions by referring to her expectations?

Another fruitful avenue for future work is to examine the link between probability and other emotions. Although we focused on children’s inferences of surprise and happiness, probability likely affects many other emotions. For instance, in skill-based games, people may feel prouder about winning when their odds of winning were very low, and they might likewise feel more ashamed about losing if their success seemed all but guaranteed. Probability also affects fear. For example, people worry more about a risk when they believe it is more likely to happen (e.g., Baron et al., 2000; Peters et al., 2006) and children may likewise recognize that risks are more worrisome when they are plausible rather than wildly improbable (e.g., Lagattuta & Sayfan, 2013).^5^

Future exploration is also needed to better understand the developmental progression of children’s probability-based inferences of emotion. For example, we do not yet know why probability is used to infer happiness at age 5, but only used for surprise at age 6 or 7. One possibility is that some inferences require more sophisticated forms of probabilistic reasoning than others. On this view, development may be constrained by children’s understanding of probability itself (for a review, see Denison & Xu, 2019) or by executive demands required to relate probabilities to one another (i.e., which may be more taxing when inferring surprise than when inferring happiness). A second possibility is that probability-based inferences depend on theory-like generalizations connecting probability with different emotions (e.g., *people are surprised when actual outcomes are much less probable than alternatives*), and that some generalizations are more difficult to acquire. A related further possibility is that such differences result because probability transforms children’s understanding of emotions, and this conceptual change may be more challenging with some emotions than others.

## CONCLUSION

In sum, children increasingly use probability to infer people’s emotions. Before coming to use probability, children infer emotions by using scripts and theory of mind. But crucially, probability-based inferences are a separate means of inferring emotions, distinct from (though compatible with) using scripts and theory of mind. Moreover, considering probability may be critical in determining how much weight children give to counterfactual alternatives to reality.

## Notes

^1^ Our focus is on how children use information about the probability of outcomes (both actual outcomes and counterfactual alternatives) to infer other people’s emotions. Probability and statistical information also play other roles in children’s emotional development. For example, infants can use the transitional probabilities within a sequence of interacting emotional faces to extract the statistical structure of the sequence (Mermier et al., 2022). For recent reviews of these other ways statistical information matters for children’s emotional processing, see Plate et al. (2022), Ruba et al. (2022), and Walle et al. (2022).^2^ The figure focuses on theory of mind and probability, because as we discuss below, both are incorporated into children’s theory of emotions, whereas scripts are independent from intuitive theories.^3^ Although people hold beliefs with differing degrees of certainty, most work on children’s “theory of mind” has not investigated whether children understand this. Instead, most work has focused on children’s grasp of situations where other people hold beliefs (whether true or false) with certainty. The few studies that examined whether children understand that other people can hold beliefs with differing degrees of certainty find that children struggle to understand this before ages 8 or 9 (Pillow, 2002; Pillow & Anderson, 2006; Pillow & Pearson, 2012; Pillow et al., 2000). For example, while most 4-year-olds judge that someone who looked at a toy and someone who did not look at a toy would be equally certain of the toy’s color, by age 8, children judge that someone who looked at the toy would be more certain of its color than someone who guessed (Pillow et al., 2000).^4^ Young children’s sensitivity to probability and base-rates in these experiments may seem surprising, given the rich experimental literature documenting adults’ failures to consider probabilistic information in their judgments and decision-making (Kahneman, 2011). However, the experimental paradigms in the infant and child experiments differ in many ways from those where adults struggled most, and some work suggests adults often succeed when tested with more comparable methods (Schulze & Hertwig, 2021).^5^ The relation between fear and probability is complicated, though, as people’s assessments of the likelihoods of some events are wildly inaccurate and fearing an event can make people overlook the fact that the likelihood of it happening is low (e.g., Sunstein, 2002, 2003).
